# New avenues in cardio-oncology

**DOI:** 10.18632/aging.101817

**Published:** 2019-02-06

**Authors:** Valentina Sala, Mingchuan Li, Alessandra Ghigo

**Affiliations:** 1 Department of Molecular Biotechnology and Health Sciences, Molecular Biotechnology Center, University of Torino, 10126 Torino, Italy

**Keywords:** anthracycline cardiotoxicity, mitochondrial DNA, autophagy, TLR9, PI3Kγ

Increased incidence of cardiac events in cancer survivors is emerging as the inevitable consequence of the use of more and more aggressive anticancer therapies. The major culprits are anthracyclines, like doxorubicin (Doxo), which, despite being cardiotoxic, remains one of the most effective antineoplastic drugs and a cornerstone in the treatment of different tumors, including the prevalent form of breast cancer.

Albeit anthracycline-induced cardiotoxicity (AIC) was initially identified as an irreversible condition, recover is now considered possible if therapy is started at the early onset of disease (Cardinale et al, 2015). Unfortunately, AIC is most frequently detected months or years after exposure to chemotherapy, making cardiac injury difficult to be timely diagnosed and treated. Even in the case of an early diagnosis, effective therapeutic options are limited. Results of randomized clinical trials with the backbone of heart failure treatment, angiotensin-converting enzyme inhibitors and beta blockers, are discordant. Controversies also emerged from the clinical use of the iron chelator dexrazoxane, the only drug approved by FDA for preventing AIC in pediatric patients, highlighting the urgent need of developing cardioprotectants that do not interfere with the anticancer properties of chemotherapy.

The simplest explanation for the cardiotoxicity of anticancer drugs is that these molecules target signaling pathways that are pivotal for the survival of not only cancer cells, but also of cardiomyocytes. Therefore, the identification of molecular mechanisms common in cancer and heart disease is the ambitious challenge of contemporary Cardio-Oncology. On these grounds, we demonstrated that phosphoinositide 3-kinase gamma (PI3Kγ) can be a common therapeutic target in AIC and cancer [1]. Although inhibition of PI3Kγ was already reported to protect against pressure overload-induced heart failure [2], its involvement in AIC was unknown. We now demonstrate that PI3Kγ, and the downstream Akt/mTOR signaling, is upregulated in cardiomyocytes after Doxo treatment, both in mice and in humans, and drives a maladaptive response that relies on inhibition of autophagy. Accordingly, genetic and pharmacological blockade of PI3Kγ protects mice against AIC by boosting the autophagic removal of cardiac mitochondria damaged by Doxo. Our study demonstrates for the first time that PI3Kγ is activated at autolysosomes by the mitochondrial DNA (mtDNA) released from damaged organelles, *via* the intracellular DNA sensor Toll-Like Receptor 9 (TLR9). These findings further support the idea that, by acting at the crossroad of autophagy and inflammation, mitochondrial damage and the associated release of mtDNA may have a broad pathogenic role [3], also including cardiovascular disease.

In AIC, we show that mitochondrial damage is the key driver of a metabolic rewiring of cardiomyocytes that, although being compensatory in early phases, fails to sustain cardiac function in the long term and drives heart failure. This model also provides a likely explanation to the still unresolved question of why AIC usually manifests long after completion of the therapy. Of note, PI3Kγ inhibition prevents the maladaptive metabolic remodeling induced by Doxo in cardiomyocytes, but whether this approach can guarantee long-term protection was not investigated and awaits further studies. Of particular interest is the finding that the metabolic shift of Doxo-stressed hearts resembles that of highly proliferating cells, like tumor cells, suggesting that one may learn from how the tumor adapts its metabolism to predict the changes that occur in cardiac cells in response to stress [4]. In line with this idea, metabolomics is emerging as an interesting tool in the diagnosis, prognosis and management of cardiovascular disease [5], and can be potentially applied to AIC, as demonstrated for instance by a clinical trial testing the effects of the anti-diabetic drug metformin in a context of Doxo toxicity (NCT02472353). Overall, the identification of metabolic fingerprints in early stages of the disease may allow early diagnosis and the development of personalized therapeutic strategies, especially for the subset of patients who are still asymptomatic but may be at risk of developing AIC.

Independently from chemotherapy, metabolism can underlie the tumor-to-heart communication in cancer patients. It is well established that the tumor can release circulating metabolic messengers, the so-called oncometabolites, that ultimately redirect cardiac metabolism. Another example is the finding that cancer causes a depletion of systemic insulin that not only contributes to tumor growth, but concomitantly impairs cardiac insulin signaling, leading to heart failure [6]. A further layer of complexity in the communication between the tumor and the heart is the bidirectionality. The emerging view is indeed that the failing heart itself may promote cancer [7,8], though the underlying mechanisms are still largely obscure, and the involvement of metabolism is undoubtedly worth investigating. Overall, these studies suggest that cancer and heart failure should be considered and treated as comorbidities.

**Figure 1:**
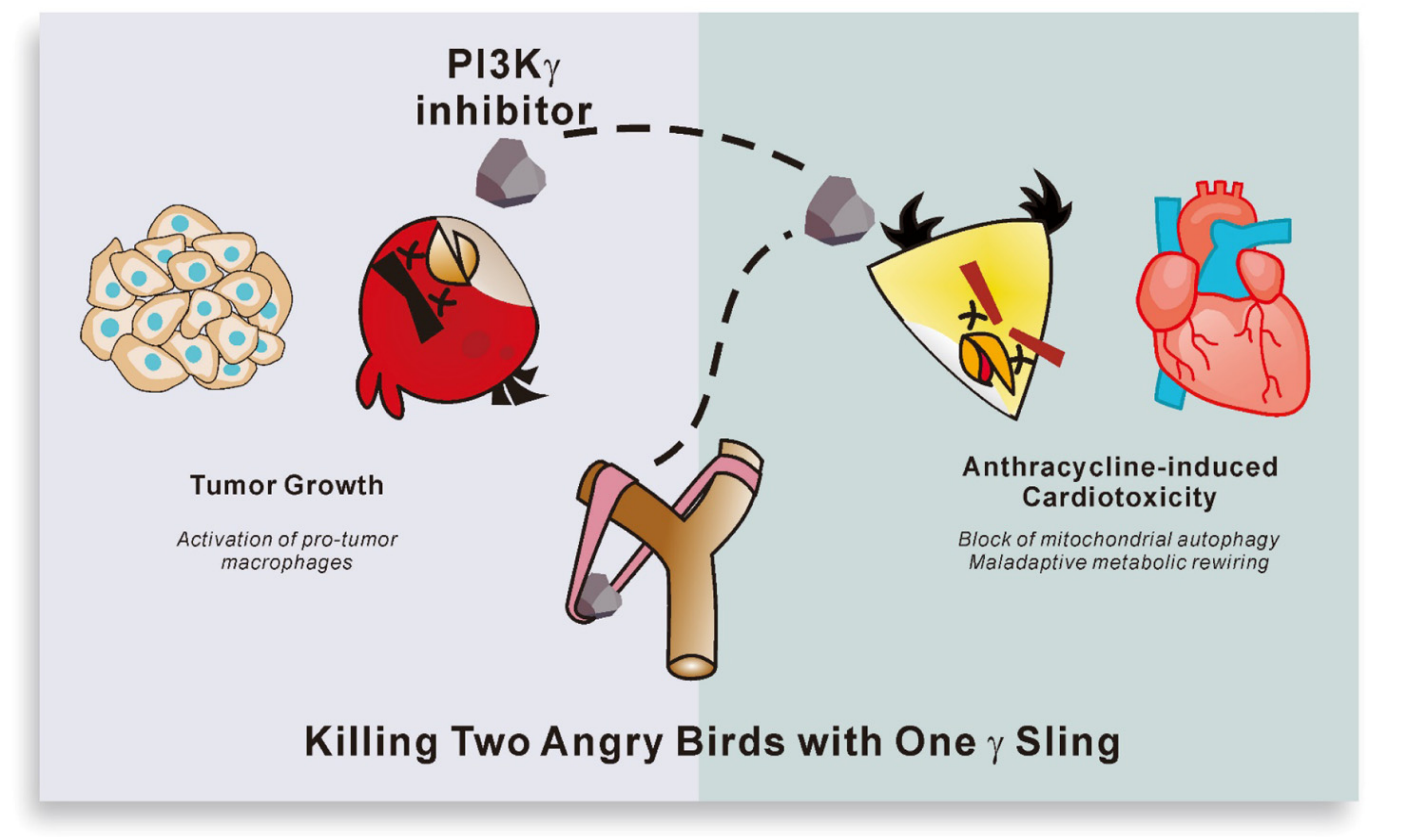
**Dual action of PI3Kγ inhibitors in AIC.** PI3Kγ inhibition protects against anthracycline-induced cardiotoxicity by boosting autophagic clearance of damaged mitochondria and preventing a maladaptive metabolic rewiring of cardiomyocytes. Furthermore, PI3Kγ blockade synergizes with the anticancer action of anthracyclines, and delays tumor growth by activating specific subsets of tumor-associated macrophages. Overall, PI3Kγ inhibitors may be the stone to simultaneously kill the two angry birds of cardiotoxicity and cancer.

Whether PI3Kγ contributes to the bidirectional crosstalk between heart and tumor is still an open question. Yet, evidence accumulated so far indicates that PI3Kγ contributes to maladaptive cardiac remodeling in response to various stresses and promotes tumor growth (Kaneda et al, 2016). Accordingly, we show that both genetic and pharmacological blockade of PI3Kγ synergizes with Doxo to delay tumor growth and concomitantly protect the heart of tumor-bearing mice from Doxo cardiotoxicity. Hence, our findings pinpoint PI3Kγ as an essential element at the crossroad of cancer and heart disease. Intriguingly, PI3Kγ inhibitors like IPI145 and IPI549 are already available and under clinical evaluation as anticancer agents, suggesting that PI3Kγ inhibition is feasible and may be the sling to “kill two angry birds with one stone” in cancer patients (Figure 1).
